# Palliative care nurse champions’ views on their role and impact: a qualitative interview study among hospital and home care nurses

**DOI:** 10.1186/s12904-021-00726-1

**Published:** 2021-02-18

**Authors:** Marijanne Engel, Lia van Zuylen, Andrée van der Ark, Agnes van der Heide

**Affiliations:** 1 Department of Public Health, Erasmus MC, University Medical Center Rotterdam, Dr. Molewaterplein 40, 3015 GD Rotterdam, The Netherlands; 2 Department of Medical Oncology, Amsterdam University Medical Centers, De Boelelaan 1117, 1081 HV Amsterdam, The Netherlands

**Keywords:** Palliative care, Hospitals, Home care services, Nurses, Generalist palliative care, Specialist palliative care, Interview

## Abstract

**Background:**

One of the strategies to promote the quality of palliative care in non-specialised settings is the appointment of palliative care nurse champions. It is unclear what the most effective model to implement the concept of nurse champions is and little is known about palliative care nurse champions’ own views on their role and responsibilities. This paper aims to describe views of palliative care nurse champions in hospitals and home care on their role, responsibilities and added value.

**Methods:**

In 2018, a qualitative interview study was conducted with 16 palliative care nurse champions in two hospitals and four home care organisations in the southwest of the Netherlands. The framework approach was used to analyse the data.

**Results:**

Most palliative care nurse champions described their role by explaining concrete tasks or activities. Most nurse champions perceive their main task as disseminating information about palliative care to colleagues. A few nurses mentioned activities aimed at raising awareness of palliative care among colleagues. Most nurses were to a limited extent involved in collaboration with the palliative care expert team. Hospital nurse champions suggested that more support from the palliative care expert team would be helpful. Most nurse champions feel little responsibility for organisational tasks and inter-organisational collaboration. Especially hospital nurses found it difficult to describe their role.

**Conclusion:**

The role of palliative care nurse champions in hospital and home care varies a lot and nurses have diverging views on palliative care in these settings. Comprehensively fulfilling the role of palliative care nurse champion is a challenge. Careful selection, training, support and task descriptions for nurse champions are needed to make the concept of nurse champions work in palliative care.

**Supplementary Information:**

The online version contains supplementary material available at 10.1186/s12904-021-00726-1.

## Background

Palliative care is an approach that improves the quality of life of patients with an advanced incurable illness and their families. Palliative care is mostly offered in non-specialised care settings by healthcare professionals for whom palliative care is not their main expertise [1–3]. One of the strategies to promote the quality of palliative care in non-specialised settings is the appointment of palliative care nurse champions [1–5].

The concept of nurse champions or link nurses is applied to a range of nursing specialties, such as infection prevention and control [6–11], tissue viability [12, 13], diabetes [14, 15] and palliative care [1, 3–5, 16–18]. The roles and responsibilities of nurse champions vary greatly [7]. Sometimes they are mainly expected to disseminate knowledge on their focus area to colleagues [7]. In other cases they are expected to raise awareness of patients’ specific care needs [1, 4] or to have a leading role in the implementation of innovative care interventions [19]. Nurse champions often collaborate in a network [2, 7, 19]. It is unclear what the most effective model for the concept of nurse champions is [3, 7]. Both in hospitals and in primary care, the model of palliative care nurse champions has been found to have much potential for contributing to high-quality palliative care [1]. In recent literature authors emphasise the potentially important role of palliative care nurse champions in adequate use of generalist palliative care versus specialist palliative care [1, 4, 20]. Other studies found that in hospital and in home care the palliative care nurse champion (in studies on home care also referred to as specialised palliative care community nurse) provides direct clinical care and also has an important role in transferring knowledge to other healthcare professionals [2, 21]. However, several authors state that the role of palliative care nurse champion in both care settings needs more clarification, including in what phase in the illness trajectory and for what reasons they should be involved in patient care, and what their role is in relation to the palliative care expert team [1, 21]. Whereas the majority of patients with an advanced incurable illness are at least once transferred between different care settings [22], palliative care nurse champions may also play an important role in promoting adequate transfers and inter-organisational collaboration [23–26].

Several studies found barriers to the effectiveness of nurse champion programs: nurse champions may fail to attend meetings due to a high work load [4, 27], be unable to disseminate knowledge to fellow staff [27] or have insufficient skills or experience for the position of nurse champion [4, 27]. Little is known about palliative care nurse champions’ own views of their role and responsibilities. The aim of this study was to describe the views of palliative care nurse champions in hospitals and home care on their role, responsibilities and added value. Therefore, we studied the following research questions:

1. How do palliative care nurse champions in hospitals and home care perceive and fulfill their role and responsibilities?

2. What do palliative care nurse champions in hospitals and home care need to adequately fill in their role and responsibilities?

3. How do palliative care nurse champions perceive their role and responsibilities in inter-organisational collaboration and transfers of patients?

## Methods

This interview study was part of a larger study on quality of palliative care in the Southwest Region of the Netherlands. We applied an explorative qualitative research approach using semi-structured interviews to assess palliative care nurse champions ‘lived experience’ of their role and responsibilities [28]. We conducted individual interviews in order to explore individual views and experiences.

### Setting and participants

Two hospitals expressed their interest in participating in this study. In order to gain insight into the role of palliative care nurse champions in a diversity of care settings, we also asked other care organisations to participate. These care organisations differ in the type of care provided (only home care or also residential care), size of the work area and organisational model. Convenience sampling was used in different care settings that were interested in participating in the study. Participants were nurses who had the role of palliative care nurse champion within an inpatient ward in one of two hospitals or one of four care organisations, one organisation only providing home care, the other three also providing care in care homes and nursing homes. All palliative care nurse champions in the two hospitals (*n* = 9 and 12, respectively) were approached directly by email, palliative care nurse champions in the four care organisations were invited by local contact persons (i.e. *n* = 1 to 4 nurse champions per organisation). Sixteen palliative care nurse champions agreed to be interviewed: five nurses working in one hospital and five in the other hospital, and six home care nurses working in four different care organisations. The interviews were conducted in participants’ workplace. Interviews in hospital lasted an average of 30 min and in home care organisations an average of 45–60 min. The palliative care nurse champions were all Registered Nurses, but had varying experience in palliative care. Seven nurse champions had completed extra training in palliative care at ISCED level 4 or 5. In the Netherlands, for the nursing specialty palliative care this is usually a 1 year course. In this course nurses learn to adjust interventions to the palliative care needs of patients and their loved ones; to provide information and advice to patients on a wide range of questions and uncertainties, and to deal with complex situations; to collaborate in a multidisciplinary team and provide advice to colleagues on complex care needs. Once nurses have completed this course they may call themselves palliative care nurses, although this is not a formal qualification in the Netherlands. Further, three nurses had completed a basic training in palliative care and six nurses had not followed extra training in palliative care (Table 1).

**Table 1:** Characteristics of respondents

Characteristics	Number of respondents (*n* = 16)
Gender	
Women	16
Work setting	
Hospital	10
Cardiology and neurology ward	1
Cardiovascular ward	2
Coronary Care Unit and Emergency department	1
Oncology/hematology ward	3
Pulmonology ward	2
Urology ward	1
Home care organisation	6
Profession	
Registered nurse	16
Training in the field of palliative care	
Extra palliative care training at ISCED level 4 or 5 ^a^	7
Basic training of one to three (half-)day courses, or e-learning	1
Minor in palliative care during nursing education	2
No extra training in palliative care	6
Years working as palliative care nurse champion	
< 1 year	2
1–5 years	7
6–10 years	4
> 10 years	3

^a^ *ISCED* International Standard Classification of Education: level 4 or 5 refers to higher professional (nurse) education

### Data collection

The interviews were conducted from May until November 2018. We developed an original topic guide to structure the interviews. Topics included participants’ perspectives on their role and responsibilities as palliative care nurse champion, how they perceived their collaboration with the palliative care expert team and their experiences with inter-organisational collaboration in palliative care (see Supplementary file 1). Data were collected by ME.

### Data analysis

Interviews were audio-recorded and transcribed. During and after the interview also field notes were made. The data were analysed using the principles of the Framework approach, a modified form of thematic analysis that is designed to provide a systematic and transparent approach to data management and analysis [29]. Line-by-line coding of each transcript was completed using the software program NVivo 12. Codes were grouped in categories and underlying themes. We used a deductive approach, using an initial framework drawing on existing literature about the role of palliative care nurse champions. This initial framework consisted of the following elements: role in clinical practice, role in relation to the palliative care expert team, role in inter-organisational collaboration. The process of constant comparison was undertaken so that the early stages of analysis informed subsequent data collection and we would find additional information throughout the research process where possible. For example, we coded answers referring to their care for patients having palliative care needs in their ward. Subsequently, we identified subthemes related to their role in clinical care and from these subthemes we moved to elements of their role. The field notes were used as supporting material for the analysis.

We aimed for thematic saturation which was determined by consensus of the researchers that no new themes in relation to the research questions had emerged in the last interviews. Special attention was paid to commonalities and differences between individual interviews and across settings. To enhance the trustworthiness and credibility of the data analysis, we used member checking, reflexivity and audit trail. Member checking was done by sending each respondent a summary of the transcript and asking whether this summary properly reflected the interview. Respondents’ feedback was added to the transcript. Reflexivity involved the use of memo writing and regular meetings with the research team to discuss the analysis. Audit trail involved extensive documentation of the research process.

Further, the research team all have experience in providing palliative care and/or doing research in palliative care. ME is a nurse non-practising, MSc and PhD student. LvZ is (practising) MD and professor in hospital palliative care, AvdA is MSc and junior researcher in palliative care, AvdH is MD and professor of medical care and decision making at the end of life. The researchers were and are not affiliated to any of the hospitals or care organisations involved and did not know the nurses who were approached. For reporting our findings we followed the COREQ guidelines [30].

## Results

Findings from the interviews relate to the palliative care nurse champions’ activities and responsibilities in patient care, their position in the care organisation in relation to the palliative care expert team and their role and responsibilities in inter-organisational collaboration (Table 2).

**Table 2:** Summary of palliative care nurse champions’ role

Elements of palliative care nurse champions’ role	Subtheme
Activities and responsibilities in patient care in a non-specialised care setting	˗ View on palliative care ˗ Own knowledge of palliative care ˗ Description of role, responsibility and added value in the ward/team
Position in the care organisation in relation to the palliative care expert team	˗ Accessibility and frequency of asking advice regarding a patient with one or more complex problems from the palliative care expert team in this ward/team ˗ Experiences with providing advice ˗ Position in relation to the palliative care expert team ˗ Barriers/facilitators in collaboration with the palliative care expert team ˗ Role in collaboration with the palliative care expert team
Role and responsibilities in inter-organisational collaboration	˗ Experience with inter-organisational collaboration ˗ Importance attached to inter-organisational collaboration ˗ Role in inter-organisational collaboration and information transfers ˗ Perspective on quality of nursing/medical/medication handovers for patients transferred from or to other care settings ˗ Communication with regard to patients being transferred from or to other care settings

### Activities and responsibilities in patient care in a non-specialised care setting

When they were asked about their role as palliative care nurse champion, hospital nurses reported varying numbers of patients who need palliative care in their ward. Numbers ranged from a small minority (cardiovascular ward) to about half of the patients (oncology/hematology ward), to almost all patients (pulmonology ward). They also reported that, as far as they knew, their role of palliative care nurse champion is not formalised. In general they described their role as being responsible for keeping their knowledge on palliative care up-to-date and informing colleagues in their ward about new insights and developments. According to most hospital nurses their role was either on hold or interwoven with their regular work as a nurse.

*“Because this is a lung oncology ward, you don’t really think about palliative care, because almost all we do is palliative care. If there is a really difficult case, and we start talking about it, it often turns out that we are already doing very well. When a physician wants to give a patient some chemo, we often say: that does not seem the right thing for this patient. Nurses are more often consulted. Physicians trust us in these things.” (Hospital nurse 2, trained in palliative care on International Standard Classification of Education (ISCED) level 5).*

Most hospital nurses consider palliative care to be one of many areas for which nurse champions are appointed, and they could not indicate when they had actually started being a palliative care nurse champion. They get no extra hours or salary for activities related to this role. Hospital nurses who had had training in palliative care at ISCED level 4 or 5 (three out of five in hospital one, two out of five in hospital two) reported that they felt they could do much more, for example update guidelines or provide training. Reasons for not being able to do so included a high workload and insufficient appreciation of their palliative care expertise in the hospital. Some also reported that they miss the support of the palliative care expert team.

The views of home care nurses about which patients in their practice need palliative care vary somewhat. Some nurses mentioned patients with a life expectancy shorter than 3 months, others suggested that patients with a longer life expectancy or patients who are in a stable phase of their illness (e.g. Alzheimer’s disease) may also need palliative care. As a result, the numbers of patients needing palliative care in their clinical practice ranged from one patient in 2 months to at least one patient each week.

Three out of six home care nurses reported that there is some kind of formal role description for palliative care nurse champions in their organisation and that they have a few hours per week for activities related to this role. The other home care nurses had no clear role description, but felt that their role is clear to themselves and their colleagues.

Home care nurses more than hospital nurses tended to report that they consciously chose palliative care as their focus, sometimes because of personal experiences. Two out of six home care nurses did a special training in palliative care at ISCED level 4 or 5. Sharing knowledge about palliative care within the organisation is considered a very important aspect of their task as nurse champion by most home care nurses. They also see a pioneering role for themselves. They could often clearly indicate when they had started and describe their tasks as nurse champion. These tasks included visiting all patients in the palliative or terminal phase of their illness, having a coaching role towards colleagues, participating in multidisciplinary home care meetings, visiting general practitioners to promote palliative care, providing training to colleagues. Unlike hospital nurses most home care nurses feel appreciated in their role by colleagues and by their organisation.

*“I am starting a new working group that will meet every six weeks, to improve collaboration with, for example, [name of local hospital]. And discuss information from within the network [local network palliative care] and other organisations. To distribute information and to ensure that knowledge reaches the right people. These things must come to life now.” (Home care nurse 1, did e-learning and several courses in palliative care).*

### Position in the care organisation in relation to the palliative care expert team

All hospital nurses are familiar with the palliative care expert team and most consider the team easily accessible. In one hospital four out of five nurses indicated that colleagues in their ward seldom ask the expert team for advice, because they think that they have sufficient palliative care expertise themselves.

*“We have the palliative care expert team to fall back on, to ask for advice. We are used to doing it ourselves, of course.” (Hospital nurse 2, trained in palliative care on ISCED level 5).*

In the other hospital, most nurses indicated that the palliative care expert team is regularly consulted. Some nurses indicated that they would appreciate more collaboration with the palliative care expert team, but that they were awaiting an initiative from the team.

*“I miss some kind of connection between the palliative care team and the ward. Not when it comes to patient care. [ …*.] *But in addition to discussing a specific patient I would like to talk a bit more about education, how we are doing, do we need training [...]. Especially when it concerns geriatric patients.” (Hospital nurse 6, no extra training in palliative care).*

Most home care nurses are familiar with the regional palliative care expert team, but they seldom ask the team for advice. They did not express a need for more collaboration, although some think that adding a home care nurse to the palliative care expert team could have added value.

### Role and responsibilities in inter-organisational collaboration

Hospital nurses collaborate with many different care providers within the hospital. Almost all hospital nurses reported that they seldom collaborate with care providers outside the hospital. Such collaboration may involve visiting symposia or transfer of information in patient care. Hospital nurses indicated that they do not have time for more collaboration and do not see the added value. They assume that their manager or the palliative care expert team will address potential problems in such collaboration. Almost all hospital nurses assessed the quality of the handover of information upon the transfer of patients with a limited life expectancy as adequate and complete.

*“I think that transfer nurses [in the hospital] are already doing a lot to ensure that people can go home well, by ensuring that care at home is properly arranged. And also the materials needed. And the rest is actually up to us. I believe that where there is no care at home and people are regularly admitted [to hospital], I often aim for continuity home visits. So that at least someone occasionally visits those people [In the Netherlands, in some regions, patients with a limited life-expectancy who not yet need home care, may receive ‘continuity home visits’ from trained home care nurses. These nurses monitor how the patient is doing and, if necessary, organise home care or other care in consultation with the patient].” (Hospital nurse 7, trained in palliative care on ISCED level 5).*

Home care nurses reported to collaborate with many other care providers: general practitioners (GPs), nursing specialists, physiotherapists, dieticians, occupational therapists, mental healthcare  providers, informal care providers. However, their collaboration with hospital staff is limited.

*“We have no consultations with the hospital. We sometimes go to the hospital to see if people are really terminal. But it is unclear how should we register that. Then it seems I’m even more unproductive [In the Netherlands, a visit to the hospital of a patient who is not (yet) registered for home care is not eligible for reimbursement].” (Home care nurse 5, trained in palliative care on ISCED level 5).*

The organisation and financing of care do not facilitate more collaboration. Further, home care nurses suggest that care providers in the hospital have a perspective on palliative care that is completely different from their own. They think that in the home care situation nurses look at the whole person and environment and adapt care to the needs of the patient, whereas in hospital care is determined by care providers.

Home care nurses reported that they often only receive a standard paper or digital handover from the hospital for patients who are discharged, and that they often miss information about patients’ diagnosis, prognosis, treatment plan and patients’ specific care needs. They have adapted to inadequate information handovers and often ask information they need from patients and their relatives themselves. Only one of them searched actively for collaboration with the hospital.

*“Next week I will for the first time attend a multidisciplinary meeting in [name of local hospital]. I have invited myself for this and had to insist for six weeks. It concerns a very complex patient. Otherwise the hospital will simply announce that or ask if we can take over care for this patient. I think that during the intake collaboration is already possible.” (Home care nurse 1, did e-learning and several courses in palliative care).*

A few hospital nurses indicated that they also contribute to the transfer of medical information: they encourage physicians to call the patient’s general practitioner to inform them about the patient, especially in case of a discharge just before the weekend.

## Discussion

This study shows that palliative care nurse champions vary in how they perceive and fill in their role and responsibilities. Most nurses described their role as palliative care nurse champion by explaining concrete tasks or activities. Most nurses perceive their main task to be disseminating information about palliative care to colleagues, whereas some home care nurses also mentioned raising awareness, e.g. by visiting general practitioners to promote palliative care, having a coaching role towards colleagues and promoting palliative care in general. Hospital nurses seemed to be more modest about their added value than home care nurses, which may be the result of more hierarchical relationships in hospital [31] and a greater focus on cure than in home care [20, 32].

Our findings are consistent with results from other studies, that also found that nurse champions consider passing on information and knowledge to colleagues an important strategy to influence practice [3, 5, 10, 16, 33]. Home care nurses more often than hospital nurses reported that they disseminate knowledge in a structured way, for example by providing training to colleagues. They do not seem to receive much guidance on this part of their role, although this has been demonstrated to be important [33]. In a controlled before and after study in a hospital, Witkamp et al. [4] found that the introduction of palliative care nurse champions who were trained in raising ward staff’s awareness of palliative care needs, resulted in more communication about patients’ imminent death.

In general, nurses in our study indicated limited collaboration with the palliative care expert team. Several studies emphasise the important supportive role of expert teams in palliative care nurse champion networks [1, 4]. Besides this supportive role of the expert team, nurse champions may also facilitate the role of the expert team, by promoting that care providers consult the expert team when appropriate [1, 10, 18] and by assisting the expert team in providing basic palliative care education [1, 18]. Nurses in our study did not actively engage in these activities, nor were they supported to do so.

Both hospital and home care nurses in general felt little responsibility towards inter-organisational collaboration and adequate transfer of information. Inter-organisational collaboration in palliative care is known to be complex and often non-optimal [26, 34–37]. Based on the literature [3, 4, 38] and given the complexity of inter-organisational collaboration in palliative care, one may wonder to what extent a more active leadership role in striving for better inter-organisational collaboration in palliative care may be expected from nurse champions.

Nurse champions are also expected to take up an active role in implementing guidelines or in introducing new interventions [1, 4, 38]. In our study especially hospital nurses reported that they have little or no time for such activities and do not feel sufficiently appreciated to be able to take up this role. This confirms findings from other studies where nurse champions also mentioned a high workload [4, 5, 10, 27, 31] and lack of appreciation [5, 16, 31].

The lack in taking up a leadership role may also be related to nurses’ personal characteristics, such as their level of interest in palliative care [3–5], having leadership capacities [4, 38] and their ability to be assertive and enthusiastic [3, 5].

Palliative care is a relatively complex area with many unresolved issues, such as the identification of patients who need palliative care, the assessment of care needs in different domains, multidisciplinary and inter-organisational collaboration, and the responsibilities of generalist versus specialist palliative care [39, 40]. It can be questioned whether, in this complex area, nurses are able to take up a comprehensive role as palliative care nurse champion, especially if they are insufficiently supported. In their qualitative systematic review of studies on the nurse’s role in palliative care, Sekse et al. found that the nurses’ many activities make it difficult to describe their role and responsibilities in palliative care [41]. Sekse et al. argue that there is an urgent need to clarify nurses’ role and impact in palliative care [41]. The same is true for palliative care nurse champions. Our finding that especially hospital nurses with training on ISCED level 4 or 5 indicated they feel they could contribute more than they currently do may suggest that the potential of palliative care nurse champions is not fully used. Our findings add to the existing literature that, in order to support nurse champions in contributing to high-quality palliative care, the concept of palliative care nurse champion in hospital and home care needs more clarity.

### Strengths and limitations of the study

A strength of our study is that the qualitative data obtained provide a valuable insight into the views of palliative care nurse champions on their role. Trustworthiness of the findings is increased by member’s check and reflexivity. The fact that our questions were somewhat general and that especially in the hospitals the time available for the interviews was often limited, leaving little time for asking in-depth questions, can be considered a limitation. Therefore, we cannot be sure that saturation of the information about our research questions was reached. In order to justify the number of respondents, we also assessed the information power of the interviews conducted. With regard to the items that in interaction determine the information power of a study sample [42], we assessed 1. our study aim as rather narrow; 2. the sample as highly specific; 3. the theoretical background about the role of palliative care nurse champions as establishing foundation for the findings; 4. the focus of the interviews as rather clear; 5. the analysis strategy moving from within-case analysis to gradually more cross-case analysis. As we did an exploratory study, the information power seems adequate [42] to offer new insights about the role of palliative care nurse champions in hospital wards and home care. Finally, caution is advised regarding transferability of our findings to other parts of the Netherlands and Europe because of differences in health care systems and in the training of nurses in the field of palliative care.

## Conclusion

Our findings show that palliative care nurse champions in hospital wards and home care think differently about their role and tasks. Nurses have diverging views on palliative care in these settings. Both palliative care nurse champions in hospital and home care have the potential to increase the quality of palliative care. However, because of the complexity of palliative care, the role of nurse champion may involve a great challenge for nurses. Therefore, nurse champions should be carefully selected, trained and supported, and their role should be formalised. Care organisations should properly embed palliative care nurse champions in their organisation, offer them ongoing education and ensure that they are structurally supported by the management and by the palliative care expert team.

## Supplementary Information

**Additional file 1: Supplementary file 1.** Topic guide for interviews with palliative care nurse champions in hospital and home care.

## Data Availability

The data of this study are stored at the Erasmus MC, University Medical Center Rotterdam, Department of Public Health, the Netherlands. Data are available upon reasonable request to the corresponding author.

## Abbreviations

COREQ: COnsolidated criteria for REporting Qualitative research
ISCED: International Standard Classification of Education
MD: Doctor of Medicine
NVivo: A qualitative data analysis computer software package used for qualitative and mixed-methods research.
