# Supplementary material for: Palliative care nurse champions’ views on their role and impact: a qualitative interview study among hospital and home care nurses
Source: BMC Palliat Care. 2021 Feb 18;20:34. doi: 10.1186/s12904-021-00726-1 (PMC7893717; doi:10.1186/s12904-021-00726-1)
Supplement: Supplementary file 1 — Additional file 1: Supplementary file 1. Topic guide for interviews with palliative care nurse champions in hospital and home care. [file 12904_2021_726_MOESM1_ESM.docx]

**Supplementary file 1**

**Topic guide for interviews with palliative care nurse champions in hospital and home care**

1. How would you describe your role as a palliative care nurse champion in your ward/home care team/organisation?

1. Can you tell me something about how you became involved in this position?
2. Have you completed additional training in palliative care? If so, what training, was it appropriate, do you miss knowledge or skills?
3. What are your tasks and responsibilities? (in patient care, in your team, in the organisation).

2. Can you describe the discharge process of a patient …

- (for hospital nurses) going home from your ward (so transfer to the general practitioner and home care)?
- (for home care nurses) coming home after hospital admission?

3. With which care providers outside your hospital ward/home care team do you regularly collaborate?

1. What does this collaboration look like? With whom, how often, about what, are there fixed agreements?
2. What are your experiences with that collaboration?
3. How is your role as a palliative care nurse champion when collaborating with other care providers different from the role of colleagues?

4. Is there a difference in collaboration between health care professionals regarding patients with a limited life-expectancy and patients not having a limited life-expectancy? Is there a difference in information handover after patient‘s discharge from hospital to home?

5. About the quality of nursing information handovers:

- (for hospital nurses) In general, do you have the impression that the recipient (general practitioner, home care nurses) is sufficiently informed so that care can be continued properly? Do recipients ever contact you for clarification?
- (for home care nurses) In general, do you, when a patient with palliative care needs is discharged from hospital to home, receive sufficient information to be able to continue care properly? Do you contact hospital nurses if you have any questions or need for clarification?

6. Is the palliative care expert team easily accessible for you? Is the palliative care expert team ever consulted in your ward/team? If so, what are your experiences with such consultations and the advice provided?

7. When would you contact the palliative care expert team? Do you have meetings with the palliative care expert team?

8. Can you tell me about the preconditions for fulfilling your position as palliative care nurse champion: available hours, management, training?

9. What is going well in the collaboration with healthcare providers within/outside your ward/team (also with other organisations) regarding patients with palliative care needs? Where is room for improvement?

10. How do you perceive your role as palliative care nurse champion in patient care? (in care of your patients, but also your role more generally in the ward/your team, your role with regard to information exchange, training of colleagues). What do you miss/need to properly fulfil your role?
